# Valorization for Biodiversity and Ecosystem Services in the Agri-Food Value Chain

**DOI:** 10.1007/s00267-023-01860-7

**Published:** 2023-07-26

**Authors:** Ariane Voglhuber-Slavinsky, Nahleen Lemke, Joseph MacPherson, Ewa Dönitz, Mathias Olbrisch, Philipp Schöbel, Björn Moller, Enno Bahrs, Katharina Helming

**Affiliations:** 1 Fraunhofer Institute for Systems and Innovation Research ISI, Competence Center Foresight, Breslauer Straße 48, 76139 Karlsruhe, Germany; 2 Institute of Farm Management, University of Hohenheim, Schwerzstraße 44, 70599 Stuttgart, Baden-Württemberg Germany; 3 Leibniz Centre for Agricultural Landscape Research (ZALF), Eberswalder Straße 84, 15374 Müncheberg, Germany; 4 Chair of Public Law, Administrative, European, Environmental, Agricultural and Food Law, Prof. Dr. Ines Härtel, European University Viadrina Frankfurt (Oder) | Research Center for Digital Law, Große Scharrnstraße 59, 15230 Frankfurt (Oder), Germany; 5 Faculty of Landscape Management and Nature Conservation, University for Sustainable Development (HNEE), Schickler Straße 5, 16225 Eberswalde, Germany

**Keywords:** Biodiversity, Ecosystem services, Valorization, Value chain, Foresight, Private valorization options

## Abstract

This article defines the term valorization of biodiversity and ecosystem services (BES) measures, as distinguished from their valuation, and underpins it with an assessment of private valorization examples along the agri-food value chain. Valorization incentivizes measures for promoting BES, while valuation refers to its quantification. Valuation can be a step of valorization but is not indispensable. In scientific literature, the terms valorization and valuation are often used interchangeably. In addition, there is a lack of research on private options versus conventional, public policy options. Therefore, we searched for private valorization options primarily in public sources (gray literature and websites). This led to the identification of four clusters (markets for voluntary services, labeling, and certification, environmental management/CSR, and tradable permits and quotas). Based on these clusters the options were assessed from a legal and systems dynamics perspective. In addition, the viability of selected valorization options in different future scenarios was examined. The analysis revealed a wide range of private valorization options, which in contrast to public policy options that focus almost entirely on the production stage, are spread across the agri-food value chain. Their suitability differs under different future scenarios, legal and systems conditions.

## Introduction

Biodiversity and ecosystems form the basis of a variety of essential functions that contribute to human well-being (IPBES 2019). They are considered to have value, which is why they can be described as services, or ecosystem services (ES) (de Groot et al. 2010). Biodiversity and ES, or BES, are often considered together. Their contributions to human well-being are manifold and well documented, but their role in agriculture and food production are virtually inextricable (Foley et al. 2011; TEEB 2018).

Depending on how they are managed, BES can be deteriorated or promoted. Through their promotion, we can increase or maintain BES and their benefits to humans. However, the converse is also true: deterioration of BES will lead to a decrease in their value and of their contribution to human well-being (Shapiro and Báldi 2014). In this context, the concept of ES was developed in order to make the various advantages provided by ecosystems to human well-being more visible and, by that, internalizing the economic impacts on the environment into decision making (Gómez-Baggethun et al. 2010; de Groot et al. 2002). Biodiversity is gaining attention from consumers (Lindner et al. 2021), where they receive information concerning BES predominantly through the link to the agricultural and food context (Hamm et al. 2016). In addition, the increasing number of political initiatives that include objectives to halt biodiversity loss highlights the growing interest in biodiversity in public policy (MacPherson et al. 2022).

Options to manage BES aim to affect human behavior and meet demand while acknowledging preferences for BES (Börner and Vosti 2013). These options provide instruments for diverse actors in the agri-food system to engage with environmental protection, overcoming classical market failures. (Gómez-Baggethun and Muradian 2015; Loft et al. 2015; Vatn 2014). In agri-food systems, BES are often attributed to the agricultural production stage, as biodiversity is produced and “consumed” in agricultural systems. However, a more systemic approach is needed to stabilize BES to a sustainable level without being overly reliant on public policy instruments (Bennett et al. 2015; Holt et al. 2016). Public policy instruments, e.g., state regulations such as taxes and subsidies for managing BES as developed over the last two decades, have shown to reach their limit for providing sufficient incentive for protecting BES (Simoncini et al. 2019). Agri-food system consumer preferences are not only based on economic self-interest, but display a certain level of social responsibility when engaging in voluntary market interactions (Matzdorf et al. 2014). Besides economic options addressing environmental problems, soft policy approaches such as Corporate Social Responsibility (CSR) emerge increasingly in the private sector (Mathis 2008). Awareness-raising is needed to sensitize the general public, politicians, and businesses for BES (Lienhoop and Schröter-Schlaack 2018), since pure monetary values often lack the provision of recommendations towards actions following the BES valuation (Lienhoop et al. 2015).

The aim is to move away from the agricultural production stage as the center of attention to a more holistic view of all actors involved in the agri-food system, especially focusing on the agri-food value chain stages (Voglhuber-Slavinsky et al. 2021). BES can be promoted at the production stage, but to attain an actual value for the provision of BES, it has to be connected to other stages of the value chain. By bringing together different perspectives, like those of farmers, consumers, industry, policy, and academia, potentials for cooperation along the value chain can be made visible.

In this study, we differentiate between valuation and the valorization of BES, while focusing on the latter as a mechanism that incentivizes the promotion of BES in human-nature interactions through monetary or non-monetary means. Following the agri-food system approach, we focus on BES valorization options including private economic instruments, public engagement and awareness raising approaches that enable connecting BES producers and consumers as well as other stages in the food value chain. Until today, no systematic knowledge is available about the diverse valorization option that currently exist. Additionally, understanding is missing about the future viability and legal specifics of such valorization option. This study aims to fill these gaps by pursuing the following research questions:

1. What valorization^1^ options for BES exist?
2. Which valorization options are most suitable in the future agri-food system?
3. Which legal considerations have to be taken into account in the application of different valorization options?

In the section ‘Theoretical background’, we specify the terms used in this article. In the section ‘Method’, we outline the search for valorization options, the applied legal assessment and the DPSIR concept, as well as the workshop design for evaluating the viability and robustness of valorization options. While in section ‘Results’, we present clusters of valorization options of biodiversity and ecosystem services, contextualize them in the light of the DPSIR concept and their legal aspects, as well as outline their viability and robustness using future scenarios. The last two sections, ‘Discussion’ and ’Conclusion’, the discussion and conclusion are presented.

## Theoretical Background

### Instruments and Options for Promoting BES

Environmental governance is facilitated by diverse institutional arrangements, ranging from market tools and community-based approaches, involving private and civil society actors or a combination of these, alongside the underlying principles of governmental command-and-control mechanisms (Muradian and Rival 2013; Sattler et al. 2018; Vatn 2014). Sterner and Coria (2013) extend the classic deviation of markets vs. command and control policy instruments to better organize the diversity of approaches into: environmental regulations, market use, market creation, and public engagement.

For a clearer distinction in the context of our study, we follow the definition of Hahn et al. (2015), using the term “economic instruments” for those options that provide monetary incentives. However, as stressed by Muradian and Rival (2013), it is the integration of community-based and economic instruments, including private and civil society actors, that allows environmental governance to be effective. Economic instruments, such as taxes or tradable permits and quotas, can complement regulations in place as well as public engagement-based approaches (Sattler et al. 2018; Sterner and Coria 2013).

In the following context of the agri-food system and the inclusion of BES as “produced” goods and services, we focus on the role of economic instruments that use a certain degree of price signals (incentives)(Hahn et al. 2015), public engagement and awareness raising options to promote environmental change. For BES to be acknowledged and improved, measures have to be awarded, or valorized. Such valorization options should not only be feasible in the present, but also adaptable to different future developments, which can help establish a strategic orientation towards achieving sustainable outcomes.

### Valuation and Valorization

In the literature, the delimitation of the terms valuation and valorization of BES are not always clear. Valuation refers to assessing the worth or importance of BES using biophysical, socio-cultural, or monetary methods. Economic values are believed to assist an effective management of biodiversity and ecosystem services by making their value tangible to the public through translation into monetary terms (Laurans et al. 2013), which can be based either on market prices or non-market behavior (Atkinson et al. 2014).

Going beyond valuation, valorization directly or indirectly promotes BES through the connection of actors along the value chain in an interactive process (van Drooge and de Jong 2016). In a more general definition, “valorization is the process of creating value from knowledge by making knowledge suitable and/or available for economic and/or societal use and translating that knowledge into competitive [...] products, services, processes and entrepreneurial activity” (van Drooge and de Jong 2016). Kehl and Sauter (2014) state that valorization goes beyond the mere demonstration of values and monetary valuation. In our article, the term valorization is used in its widest sense, taking into account nature-oriented offers and incentives based on a non-monetary valuation as described by Wolff and Gsell (2018c).

**Fig. 1:**
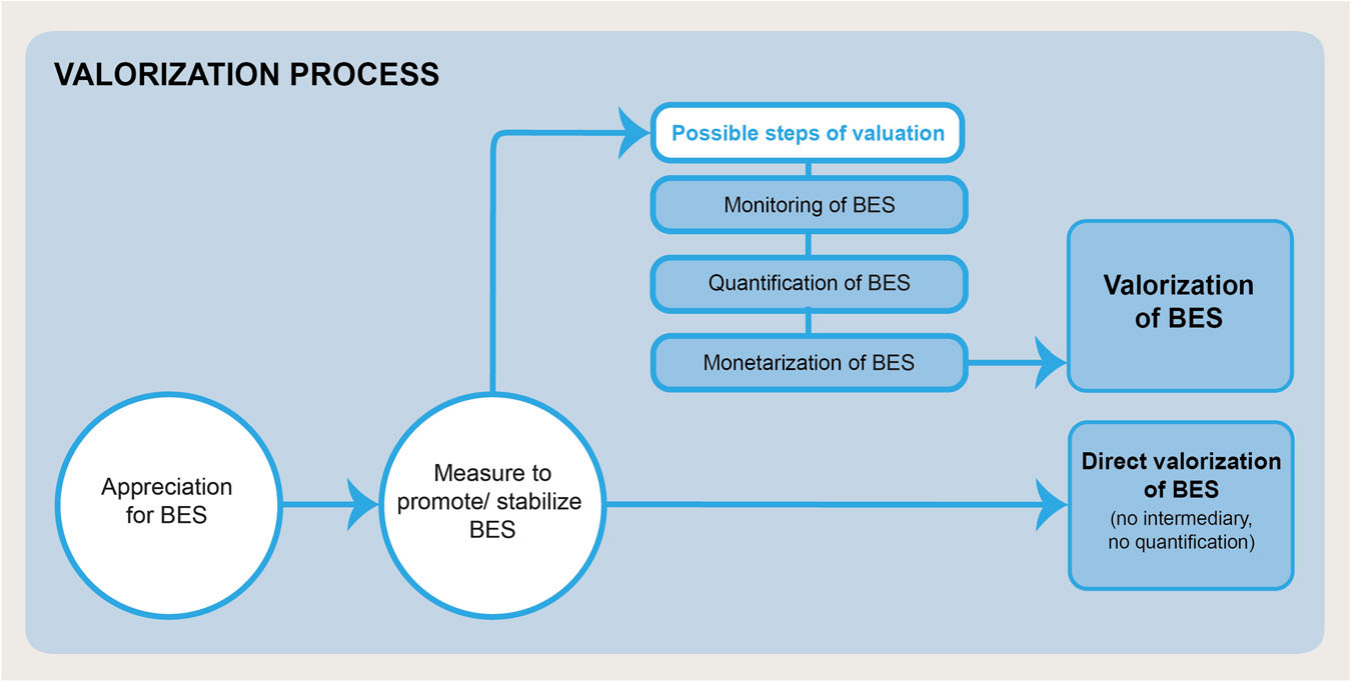
Delineation of the terms valuation and valorization

To operationalize our BES valorization concept, we understand valorization as a measure to promote and/or stabilize BES either directly or through the intermediate step of valuation (Fig. 1). Thereby, valuation is a possible, but not a necessary step towards valorization of BES.

## Method

**Fig. 2:**
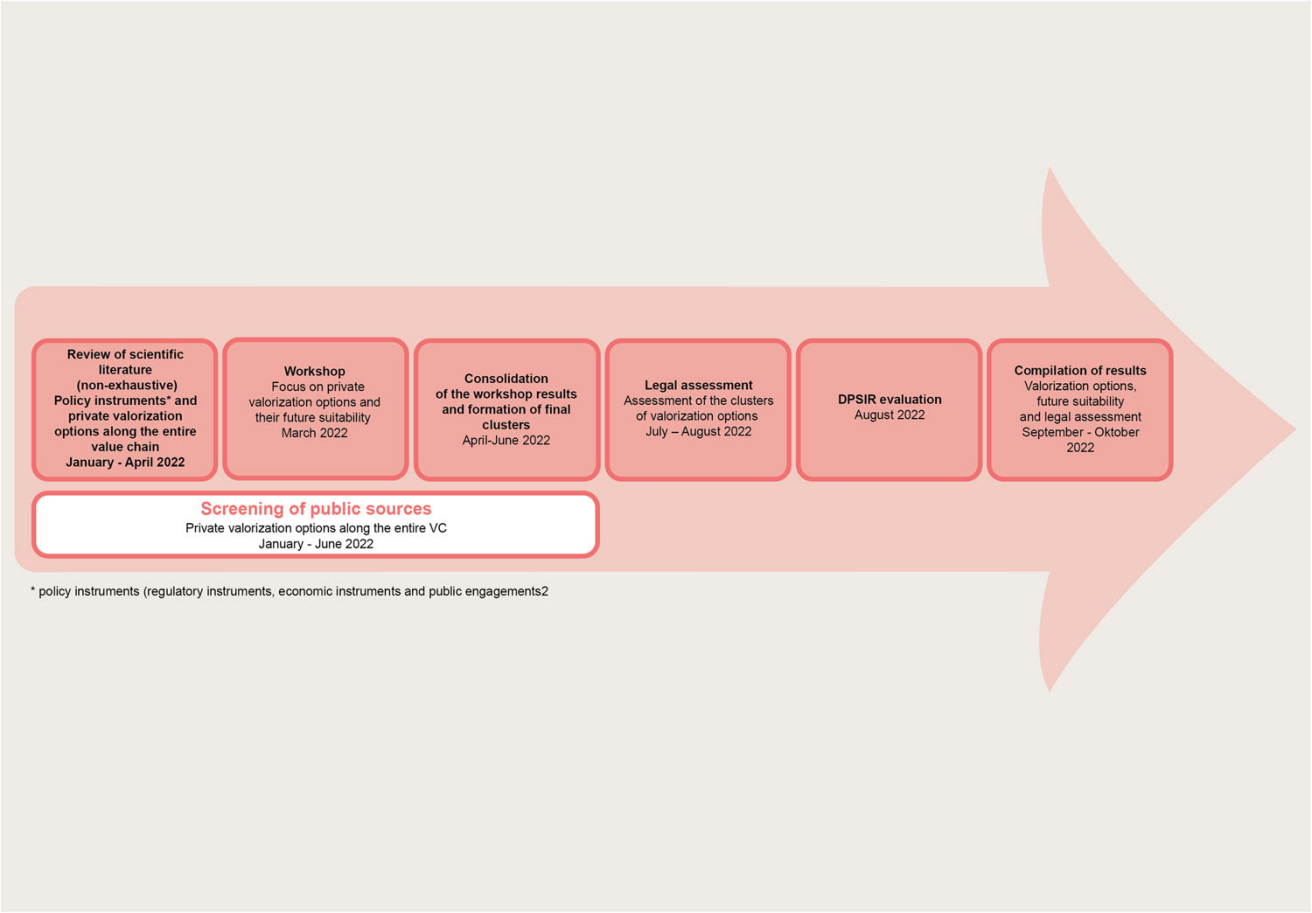
Working steps and methods used for this study

This study is based on a multi-method approach, employing literature analysis, stakeholder consultation, legal analysis, DPSIR based causality assessment and a viability check towards futures framework conditions to evaluate BES valorization options (see Fig. 2). A literature analysis was applied for conceptual purposes and to embed our research aim in the broader scientific context. In addition, a list of valorization options was elaborated using publicly available sources. In a second step, the collected options were discussed and supplemented in a workshop connecting them to the different stages of the agri-food value chain (see Fig. 4 and Appendix B), where in addition, their future viability was assessed by a group of key stakeholders from the agri-food system. In a third step, we complemented the analysis with a legal assessment of BES valorization options, assessing consequences for the involved parties and possible legal framework conditions required to implement those options. Finally, to analyze the options in a larger systems context analysed how the options impact the appreciation of BES applying the causality cycle of the *DPSIR* approach (Smeets and Weterings 1999).

### Literature Analysis and Screening of Public Sources for BES Valorization Options

**Fig. 3:**
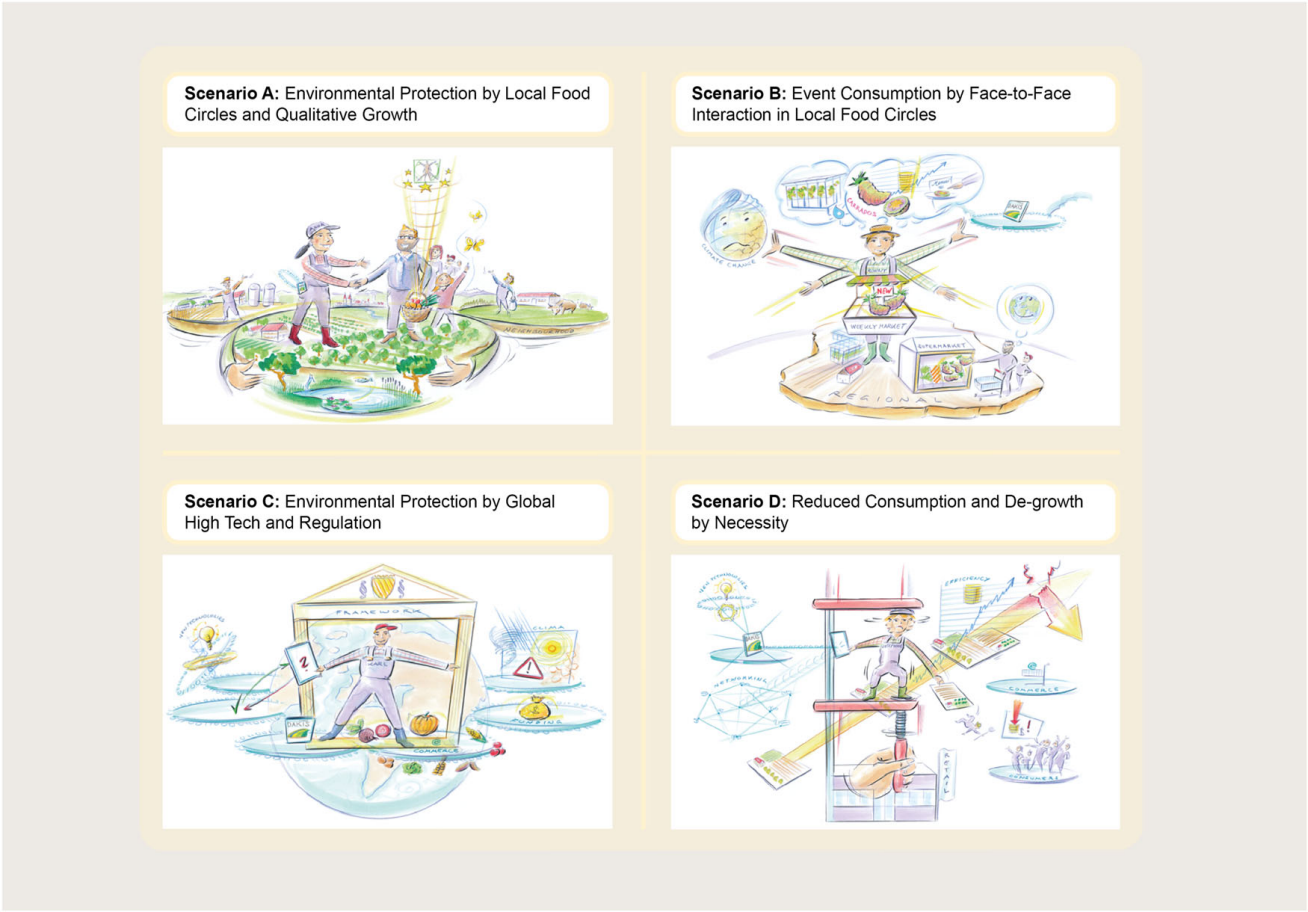
Future scenarios “Agribusiness in 2035 - Farmers of the Future” (Dönitz et al. 2020)

We conducted a non-exhaustive review of the literature concerning BES valorization in scientific and publicly available data bases and search engines. The literature databases Scopus and the evaluation algorithm KATI (Fraunhofer Institute for Technological Trend Analysis INT 2022), which is based on Web of Science, were used for the scientific search. As the terms valorization and valuation are not clearly differentiated, the following search terms were applied for both databases: valorization, valuation, commodification, ecosystem, service, biodiversity, agri-food, value chain, supply chain, LCA, future, and their respective combinations. For the conceptual work on the definitions of valorization, a worldwide view of publications was adopted. The search was conducted between January and April 2022. The preliminary review of the literature from the fields of agriculture and BES revealed a striking lack of documentation of private valorization options. This could be a result of unclear terminology regarding valorization and valuation (see section ‘Theoretical background’ background) or the fact that private valorization options are rapidly emerging and highly diverse, and therefore the theoretical basis has not been laid out for them yet. Screened literature revealed a focus of scientific articles on public policy instruments for the valorization of BES. We, therefore, extended our analysis to non-scientific sources. This was done with a screening of non-scientific, publicly accessible websites promoting or explaining valorization options. We searched for options in industrialized countries with a focus on Germany, Austria, Switzerland and the European Union as a whole. These geographical confines enabled us to integrate the different valorization options into one legal context, as well as into the future scenarios (see Fig. 3) used for the viability check.

The valorization options were clustered into four categories of valorization mechanisms. The authors acknowledge that it is not an exhaustive summary of all options available, but rather a comprehensive selection of different types of valorization options.

### Workshop: Setting the Valorization Options in the Context of Future Scenarios

Measures to promote BES are ideally implemented over longer periods of time. However, changing framework conditions play a major role in the choice of certain measures and options for their valorization. Given that the agri-food system is under constant change and influenced by many technological, political, environmental and socio-economic drivers (FAO 2017; Fukase and Martin 2020; IFPRI 2020; Moller et al. 2019), a future-oriented perspective can help with anticipating and reacting to some of these changes.

Scenarios provide a basis for decision making, since they give us the strategic orientation in the future. We used scenarios developed by Dönitz et al. (2020) in the project DAKIS^2^ to assess the viability of the valorization options. For descriptions of the scenarios used in our study, please refer to Fig. 3 and the Appendix.

The multi-stakeholder approach followed within the workshop involving 31 experts was applied to integrate a broad spectrum of expertise from different professional fields and perspectives from the agri-food value chain. Participants were selected as to cover the entire agri-food value chain. In the first part of the workshop, participants were presented with the aims of the study, the scientific background, the clusters of valorization options and the four scenarios for the viability check. In the second part, participants were split into three smaller working groups, where they were asked to comment on the suggested options as well as to contribute their own ideas for options. For selected valorization options, each group assessed the viability within the four different future scenarios. This was done to provide guidance for the application of certain options within future agri-food systems (see Table 5). The leading question for each valorization option was: “Does the framework of one scenario foster or hinder the implementation of the option?” Scales from +2 (supported by the framework), +1 (slightly supported), -1 (implementation hindered) and -2 (implementation impossible) were applied for quantification. The possibility to give a value of 0 was avoided to scrutinize the options for their most important aspects. An option was deemed viable if it was at least slightly supported (e.g., +1 or +2) by the framework conditions in at least one scenario. Following the workshop, the group assessments were consolidated to derive the robustness of the options.

### Legal Assessment of the Options

In order to legally assess the BES valorization options, a short overview to clarify the use of terms is given in the following. First, the distinction between the political sphere and the genuine legal sphere has to be made. Where the legal sphere contains enforceable rules - so called hard law -, the political sphere can be considered as the societal forum in which concepts of valorization are developed (e.g., political acts as for example the EU Biodiversity Strategy^3^) (Dörr and Nachtmann 2022; MacPherson et al. 2022). These political agreements, or ‘soft law’, are not enforceable by state power, but generate strong political pressure in case of non-compliance (e.g., the Sustainable Development Goals of the United Nations). In the legal sphere, legal instruments are created by public law and private law. Public law is understood as that law which empowers public authorities as such and is to be considered as specific law of the state.^4^ In contrast, private law intends to balance the interests between private subjects, for example, by establishing rules for contractual agreements, which again operate as legal tools for certification systems and various further valorization options. In this context, we– in a first step - analyzed how the different categories of valorization options can be allocated according to the aforementioned categorical scheme. Based on the allocation of the respective options to the genuine legal sphere we – in a second step – take a closer look how these options are legally set up and how they are legally enforced.

### Assessment of the Valorization Options Using the DPSIR Approach

**Fig. 4:**
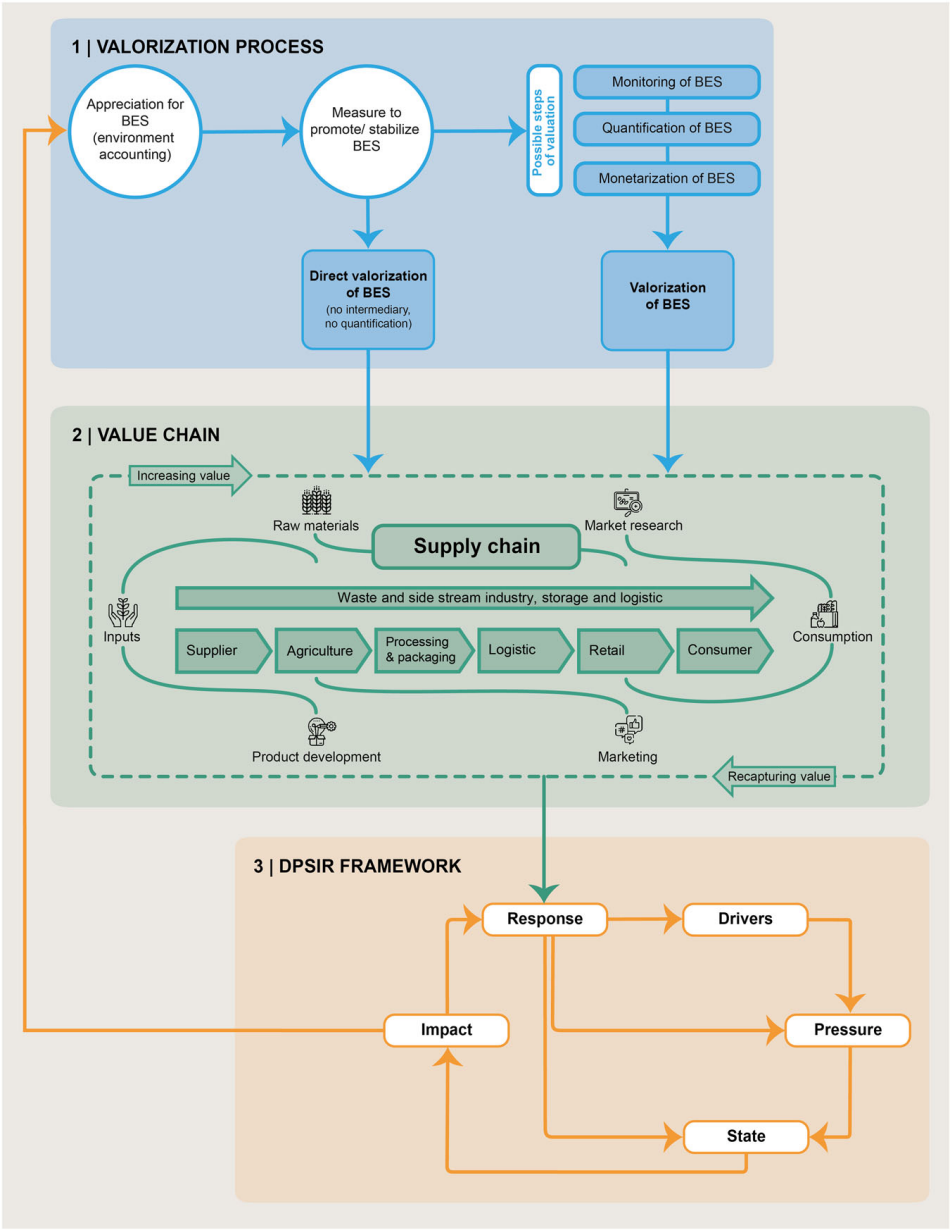
The DPSIR-valorization chain framework demonstrates the wider system interaction of BES valorization and the cyclical process of adaptation therein

To account for causal dynamics and feedbacks affecting valorization options, we integrate the DPSIR framework with our valorization and value chain approach (see Fig. 4). The DPSIR analytical tool is a systems-based approach that underlines cause-effect relationships between social, economic, and environmental system elements, which consist of *Drivers* (*D*), *Pressures* (*P*), *States* (*S*), *Impact* (*I*), *Response* (*R*) (Smeets and Weterings 1999). *Drivers* describe the developments in society, economics and demographics that influence population, technological development, international trade, and societal attitudes, which lead to *pressures*. *Pressures* are direct results of human activities, such as pollution, land use change, climate change, or overuse of resources that lead to changes in biological, physical, or chemical *States* of the environment. Environmental changes affect the provisioning of BES, which impacts societal objectives and human well-being. These *Impacts* can induce *Responses* from society aiming to reduce *Impacts* by influencing other elements in the system, through prevention, compensation or adaptation. The DPSIR framework has commonly been used for analyzing the consequences of policy-based responses, such as taxes, subsidies or regulations, but we adapt it to focus on private valorization options for BES as *Responses*.

## Results

The identified valorization options are clustered into four categories: (i) Markets for voluntary services, (ii) Labeling and certification, (iii) Environmental management/CSR and (iv) Tradable permits and quotas. The list of options reveals a rather clear focus on economic incentive-based options or those focusing on environmental awareness raising in the corporate context. Each cluster is outlined in the following sub-sections and supplemented with a legal and DPSIR assessments. A selection of valorization options and their position within the agri-food value chain is displayed. Lastly, an assessment of the viability and robustness of certain valorization options is provided.

### Valorization Options

In the following, the valorization options are presented according to their assignment to the four clusters: Markets for voluntary services, Labeling and certification, Environmental management/CSR and Tradable permits and quotas. The tables contain the options as well as the mechanism for valuation and valorization for the respective option. More details and explanations on the options can be found in Appendix B.

### Cluster: Markets for Voluntary Services

The Markets for voluntary services cluster mostly targets stakeholders already interested in the area of BES. It should be highlighted that some offers (e.g., market places for biodiversity measures) belong as well to nature protection without a connection to agricultural production. The concept of production-integrated compensation describes measures to protect or promote biodiversity “on agricultural and forestry land with continued agricultural and forestry use”. In contrast, offsetting activities as well can be implemented on agricultural land, which is then lost for production purposes (Sponagel et al. 2021).

The valuation of the biodiversity measures is either based on the assessment of the implementation of the measure, like for example with “KLIM” or “Insektenhelden”, or an assessment of the change in BES is conducted, representing results-based approaches, for example with “Agora Natura” or “Contracts 2.0”. The valorization can be expressed through a variety of approaches, like consumer payments for the implementation of the measures, as is in the options “Vielfeld” and “Insektenhelden”. These payments are connectable to the generation of certificates like for “Agora Natura”. The “Regionalwert AG” in contrast works with share purchases to valorize their contribution to the promotion of BES. “BITE” follows an awareness raising approach for the valorization by giving information on the impact on biodiversity of their meals (see Table 1).

**Table 1:** Markets for voluntary services

Valorization option	Mechanism (valuation and valorization)
CONTRACTS 2.0 (contracts2.0 2022)	valuation: result-based payments using indicators and quantification methods valorization: integration of the cost for environmental services into the product price
AGORA NATURA (AgoraNatura 2021)	valuation: quantification of biodiversity and ES valorization: contract between private companies/persons and farmers with certification
REGIONALWERT AG (Regionalwert Leistungen 2022)	valuation: value performance calculator (Leistungsrechner) valorization: share purchase for citizens (Bürgeraktie)
KLIM (KLIM 2022)	valuation: method-based approach, where implemented measures are paid for based on their potential benefit calculated applying scientific data valorization: payments from investors, consumers or public authorities
ECOCROWD (EcoCrowd 2022)	valuation: dependent on the project valorization: payment for a project connected to various rewards
FUTURE PLANTER (FuturePlanter 2022)	valuation: the implementation of the measure is done by individuals and is not assessed valorization: payment from consumers
VIELFELD (VIELFELD 2022)	valuation: payments for farmers to adopt measures valorization: payments from consumers
INSEKTENHELDEN (INSEKTENHELDEN 2022)	valuation: monitoring of measures and subsequent payments for farmers valorization: payments from consumers
BITE (WUPPERTAL INSTITUT 2017)	valuation: measurement of biodiversity valorization: information on the impact on biodiversity of different meals
MOORFUTURES (MoorFutures 2022)	valuation: scientific monitoring of the climate impact as part of the project planning and every 5 years after the implementation of the rewetting of the peatland valorization: purchase of the MoorFutures carbon certificates
CLIMATE FARMERS (Climate Farmers 2022)	valuation: monitoring valorization: voluntary carbon market for companies
TREECYCLE (TREECYCLE 2022)	valuation: each eTREE is legally linked to a real eucalyptus tree using a blockchain-driven security token valorization: voluntary investment in tree planting via tokens
BLOOMING LANDSCAPE NETWORK (NETZWERK BLÜHENDE LANDSCHAFT 2022)	valuation: no valuation of BES valorization: direct payment for a measure (measure oriented), signs and info boards

#### Legal Assessment

New contractual regimes are subject to research activities and could set impulses for new valorization models. In this context, “Contracts 2.0” intend to create a set of legal provisions that establishes a new contractual model, which integrate the costs for environmental services in the product price. In some cases, these contracts de facto lead to a transfer of money to farmers which conduct their business in a manner that protects biodiversity. From an economic point of view, such models would provide stronger incentives, if holding a certificate would have an economic value for the holder.

Registered shares are another legal option to create markets for voluntary services. Since these stocks can be purchased by customers that are interested in the regional biodiversity protecting business concept of the respective company, they can provide active financial support for a bigger initiative. This model is run by “Regionalwert AG”.

With the Taxonomy Regulation,^5^ the European Union for the first time defines in a binding manner when economic activities are considered environmentally sustainable (Lamy and Bach 2020). Both financial and non-financial companies must provide investors with information on the contribution of their economic activities to environmentally sustainable operations (Art. 8) (Dietz 2022). Further specification is set through Art. 8 (4) as the Commission shall adopt a delegated act to specify the content, methodology, and presentation of information to be disclosed by both non-financial and financial undertaking (European Commission 2021). The Taxonomy Regulation can also be significant with regard to the following clusters, particularly in the area of assessment of ecological sustainability of companies, as it provides fundamental guidelines for investments in voluntary services.

Tokens for tree planting are measure-oriented as the token is generated for the action of planting a tree, regardless of the results it generates. If the market is regulated in that way, like for CO_2_ certificates, legal acts could be adapted relatively easy in order to rearrange existing structures. In contrast, if the results of BES measures get assessed and serve as the basis for valorization, the associated legal framework has to be more specific to each way of the assessment of the results.

#### DPSIR assessment

The options within this cluster mainly affect *Pressures* by changing the behavior of producers and *States*, by conserving BES through protected areas. The options in this cluster that focus on results-based measures require a significant degree of quantification and monitoring during implementation. In this case, certification processes are needed to ensure trust between producer and consumer and create the necessary conditions for a functioning market. In the example of “Agora Natura”, an online market place allows customers to financially contribute to a variety of certified nature conservation projects, most of which concern a particular habitat or ecosystem state through establishing protected areas. *Pressures* on BES are also addressed by certain options in this cluster, as in the case of the “Regionalwert AG”, which focuses on reducing pressures on BES by creating a financial conduit between investors and companies within the agri-food sector who wish to produce organic and local food. For this option, the pressures of conventional farming on BES are mitigated.

### Cluster: Labeling and Certification

Certificates as official documentation of a certain fact and labels as information attached to a product were summarized in this category. Whereas labels^6^ are directed from Business-to-Consumer (B2C), certificates communicate certain specifications between businesses along the agri-food value chain. There was a strong reservation against labeling in the workshop and the need for public governance was pointed out. It was argued that the promotion of biodiversity must take place on larger areas and should not only be promoted solely by private initiatives. Critics in the workshop mentioned the great administrative burden of implementing labeling, questioned what percentage of additional revenue accrues to the farmer as a reward, and whether they can improve product sales due to the positive image that a label provides. Nevertheless, valorization options under this cluster can be a viable means to raise awareness for the importance of BES among a broad public audience.

An example added by workshop participants within this cluster is the initiative “larch bread” (see Table 2), which supports local and regional value chain actors. Producers of grain on fields with larch breeding habitats receive a remuneration for grain yield reduction via higher prices paid by the processing partner (miller) in addition to price adaptation along processing and sales.

**Table 2:** Labeling and certification

Valorization option	Mechanism
ECO-SCORE (Eco-Score 2022)	valuation: Life Cycle Assessment valorization: labeling
PRO PLANET-BIODIVERSITY PROJECT (REWE Group 2020)	valuation: measure-oriented approach (implementation of biodiversity promoting measures): evaluation of site-suitability for measures, measure implementation and evaluation-based on scoring system, producer certificate in compliance with scoring system (regional level) valorization: certification and Pro Planet-Label „For more biodiversity“ on REWE Group products
AGRICULTURE FOR BIODIVERSITY (Landwirtschaft für die Artenvielfalt 2022)	valuation: measure-oriented approach (implementation of biodiversity promoting measures): evaluation of site-suitability for measures, measure implementation and evaluation based on scoring system, producer certificate in compliance with scoring system (farm level) valorization: “Agriculture for biodiversity” Label on Edeka products
EU ORGANIC LOGO (European Comission Agriculture and rural development 2022)	valuation: compliance with cultivation and husbandry standards (control mechanisms included) valorization: labeling and certification
NATURLAND (Naturland 2022)	valuation: sustainability assessment valorization: labeling and certification
LARK BREAD (2022)	valuation: measure-oriented approach (implementation of biodiversity promoting measures): evaluation of site-suitability for measures valorization: label “larch bread” indicating nature conservation and regionality; higher prices for the wheat for farmers
TEST MARK OF THE BIOSPHERE RESERVE SCHORFHEIDE-CHORIN (Schorfheide-Chorin 2022)	valuation: sustainability assessment valorization: labeling and certification of products
HOW MUCH IS THE DISH? (Universität Greifswald 2020)	valuation: quantification and true cost accounting valorization: awareness raising (price tag, not executed)

Within this cluster the valuation process is often conducted by a sustainability assessment, as in the example of “Naturland”, “Test mark of the biosphere reserve Schorfheide-Chorin”, and with Life Cycle Assessment, like for the “Eco-score”. Another valuation instrument is true cost accounting, as demonstrated in the example “How much is the dish” (Michalke et al. 2022). Here, the valorization option applied is a price tag that is used for awareness raising. However, it should be highlighted that the higher ‘true’ price is not actually enforced.

#### Legal assessment

Certificates offer the advantage of reduced transaction costs for stakeholders in the production process by serving as a guarantee for compliance with specific qualitative characteristics of the product. Meanwhile, labels can be used to highlight certain qualitative characteristics of a product on the consumer market. In legal terms, these certificates and labels have to be considered as collective or individual trademarks, which can be found on the national German level and on the European Union´s level of regulation as well.^7^ The qualitative characteristics of labeled products are usually subject to license agreements which put the right to use the trademark for labeling purposes on the market under the condition that the contractual agreements of the license are complied with (Olbrisch 2022).

The use of these labels is limited by the principle of labeling integrity in order to prevent failure of market (Olbrisch 2022). The principle of labeling integrity states that the factual quality of a product, on the one hand, and the quality displayed by a label have to correspond.^8^

#### DPSIR assessment

As awareness raising mechanisms for BES, Certification and labeling options play an important role in affecting Drivers, specifically by shaping social and cultural attitudes toward BES. For the most part, labels positively influence consumer preferences for BES by making its value explicit and encouraging its appreciation in the wider public arena. This can provide a strong boon for other types of BES valorization options, but especially for creating added value for producers who work to uphold environmental standards. It is at this consumer-producer interface that certification and labeling can lead to a reduction of pressures on BES, as is in the example of the “Eco-score” label that ensures the sourcing of a product did not affect the respective habitat of endangered species. Here, consumers are made aware of how a particular measure may impact BES and producers are rewarded with a premium for promoting that BES.

### Cluster: Environmental Management/Corporate Social Responsibility (CSR)

Corporate social responsibility (CSR) is a concept that recognizes that companies exert a significant impact on society and the environment through their business practices. Companies voluntarily adopt CSR approaches as a means to improve their environmental footprint and to communicate with stakeholders (e.g., shareholders, customers, governmental institutions, business partners) via public reports that they are taking environmental issues into account in their business practices. More and more, biodiversity is included in CSR practices, either through direct mitigation of biodiversity loss or supporting activities (Wolff et al. 2018a). For the most part, CSR options evaluated in this study are aimed toward awareness raising and knowledge transfer.

Several options include tools that are used for conducting enterprise-level sustainability assessment e.g., “Biodiversity Performance Tool”, “Sustainability Assessment of Farming and the Environment (SAFE)” framework and the “Sustainability Assessment of Food and Agriculture systems (SAFA)” tool (Table 3). Here, the purpose of using such tools is for raising awareness within a company on its overall environmental, social, and economic performance. The results of such internal assessments are sometimes made public by companies through annual reports as a means for communicating their interest in BES to stakeholders.

**Table 3:** Environmental management/CSR

Valorization option	Mechanism
SHOWCASE (SHOWCASE 2022)	valuation: quantification of biodiversity and ES valorization: EU CAP 2nd pillar funding, considering labeling in the future
BIODIVERSITY IN STANDARDS AND LABELS FOR THE FOOD SECTOR (Business Biodiverstiy 2022)	valuation: defining relevant criteria valorization: improving the performance of standards and labels
ENTERPRISE BIODIVERSITY 2020 (Leben.Natur.Vielfalt 2022)	valuation: specific to each company valorization: awareness raising and networking between enterprises
BIODIVERSITY PERFORMANCE TOOL (Biodiversity Performance Tool 2022)	valuation: sustainability assessment valorization: awareness raising for the farmers and promotion of sustainable measures on farm
SUSTAINABILITY ASSESSMENT OF FARMING AND THE ENVIRONMENT (SAFE) FRAMEWORK (Trifolium 2012)	valuation: sustainability assessment valorization: awareness raising and promotion of sustainable measures on farm
SUSTAINABILITY ASSESSMENT OF FOOD AND AGRICULTURE SYSTEMS (SAFA) (FAO 2022)	valuation: sustainability assessment valorization: awareness raising and promotion of sustainable measures on farm/enterprise
BIODIVERSITY IN GOOD COMPANY (Business and Biodiversity Initiative 2022)	valuation: actions and aims are set individually by the companies, assessment depending on the options valorization: awareness raising

Other valorization options include initiatives such as “Biodiversity in good company” and “Enterprise biodiversity 2020” that provide platforms for companies and industry for exchanging, collaborating and making voluntary commitments on biodiversity issues. The initiative “Biodiversity in standards and labels for the food sector” can be understood as a CSR-supporting intermediary that develops criteria (indicators) for standards and labels for measuring impacts on biodiversity. The only option in this cluster where BES are valorized through direct compensation payments is the research project “SHOWCASE”, as it cooperates with farmers to implement biodiversity-related measures that can capitalize on 2nd pillar EU CAP funding.

#### Legal assessment

Since CSR measures can be regarded as a set of self-binding rules that communicates the company´s responsibility towards the society in numerous perspectives, those rules cannot be enforced by legal means.^9^ Nevertheless, they can cause legal consequences if the relevant company binds itself to these rules because they might be regarded as advertising communication towards the consumer and can – in doing so – potentially interfere the market in case of non-compliance with the relevant CSR codex (Alber 2021).

As far as environmental management systems take into provisions of environmental law, climate protection law and other sustainability related legal provisions, these provisions cannot be seen isolated from each other. On the contrary and implying the interlinkage of contemporary environmental management and digitally driven applications: There is a practical need to integrate the different facets of digital law as it has a different logic of balancing interests compared to agricultural law itself. With regard to related environmental management systems as a component of evolving smart farming systems, the different functional logics of the traditional agricultural law and the digital law with all its innovative regulatory instruments, have to be merged to an “agri-digital law” (2019) in order to coherently assess the legal questions arising in a complex world of vulnerability, uncertainty, complexity and ambiguity of emerging novel instruments in the field of BES (Härtel 2019). This again rises synergetic effects between the different regulative concepts and compensates the uncertainties, as well as the stakeholder’s vulnerabilities arising from the aforementioned.

#### DPSIR assessment

Options within the CSR cluster primarily affect *Drivers* by raising awareness for BES on an organizational and sectoral level. As a key element in CSR, sustainability reporting on environmental impacts allows enterprises to communicate their interest in BES to stakeholders. In this regard, integrating science-based sustainability assessments in CSR strategies can improve credibility of reporting and reinforce awareness raising. SAFA, for example, is internationally recognized in the agri-food sector, meaning the results of the assessment can be communicated to a wide group of stakeholders. In cases where enterprises include BES commitments in their CSR strategies, *Pressures* to BES may also be more directly addresses, but only if those commitments are indeed fulfilled. Valorization options like “Biodiversity in good company” and “Enterprise biodiversity 2020” promote awareness at an industry level, which can drive valorization of BES.

### Cluster: Tradable Permits and Quotas

For the given options, tradable permits and quotas relate to the individual transfer of permits or quotas and are rather quantity-based instead of price-based instrument (Helm and Hepburn 2014; Pirard 2012). A market is created for certain environmental problems to efficiently manage scarce resources. Concrete permits often relate to water trading or tradable fish quotas, or transferable development rights for land planning (Sterner and Coria 2013). In the context of BES valorization options, the principle of mandatory polluter-funded payments apply. Prominent examples are the “area agencies” in Germany, which operate at the intersection of state regulation and market-based instruments. Displayed in Table 4, the “Brandenburg area agency” is listed to exemplify the valuation and valorization procedure. The same principle applies to the example of “eco dots”, a private company that equally evaluates land quality with regards to its biodiversity potential. The quantification into so called “eco dots” is followed by the commercialization on the created market.

**Table 4:** Tradable Permits and quotas

Valorization option	Mechanism
ECODOTS (ecodots 2022)	valuation: evaluation of land quality with regards to its biodiversity potential; quantification into ecodots valorization: area compensation is payed for by project developers, “ecodots” is coordinating the compensation procedure
BRANDENBURG AREA AGENCY (FLÄCHENAGENTUR BRANDENBURG 2022)	valuation: evaluation of the land suitability for compensation measures for land purchase; full cost calculation of measure implementation valorization: “Investors legally obliged to compensate for impacting on nature and landscapes pay the Flächenagentur for areas held in reserve and any compensation measures implemented” (Matzdorf et al. 2014)

#### Legal assessment

Tradeable permits and quotas provide an alternative means of internalizing external costs and complement the polluter pays principle of Art. 191 TFEU in environmental law. These options aim to promote biodiversity conservation by encouraging voluntary compliance beyond legal requirements, making them primarily economic in nature. However, they are still rooted in legal regulations.

Whereas the structurally comparable trade system for emission certificates is based on a cap and trade mechanism constituted by public law, the system of “eco-dots” is mainly based on the instruments of private law, since the certification process behind it is generally based on private contracts between the certifying company and the certificated entity (i.e., farmer or company). These private contracts refer to the relevant legal provisions which admit the compensation of impacts of biotopes, as for example § 30 para. 3 BNatschG.^10^ In this context § 16 BNatSchG declares compensational measures can be stockpiled in pools. In this way, the compensatory measures are decoupled spatially and temporally from the natural intervention (Erbguth and Schlacke 2018).

#### DPSIR assessment

As results-based options, the possibilities of valorization that fall under this cluster primarily affect *States*. Affected *states* may include, pollution levels (atmospheric CO2 emissions), water availability, fish stocks, and ecosystems, for example. In this regard, the valorization options in this cluster, such as “Brandenburg area agency” and “eco dots”, function on the principle that protecting an ecosystem state for a particular BES is achieved through land protection and area compensation. Hence, by protecting land and the ecosystems embedded in them, *Impacts* to BES from future pressures are averted.

### Valorization Options along the Agri-Food Value Chain

**Fig. 5:**
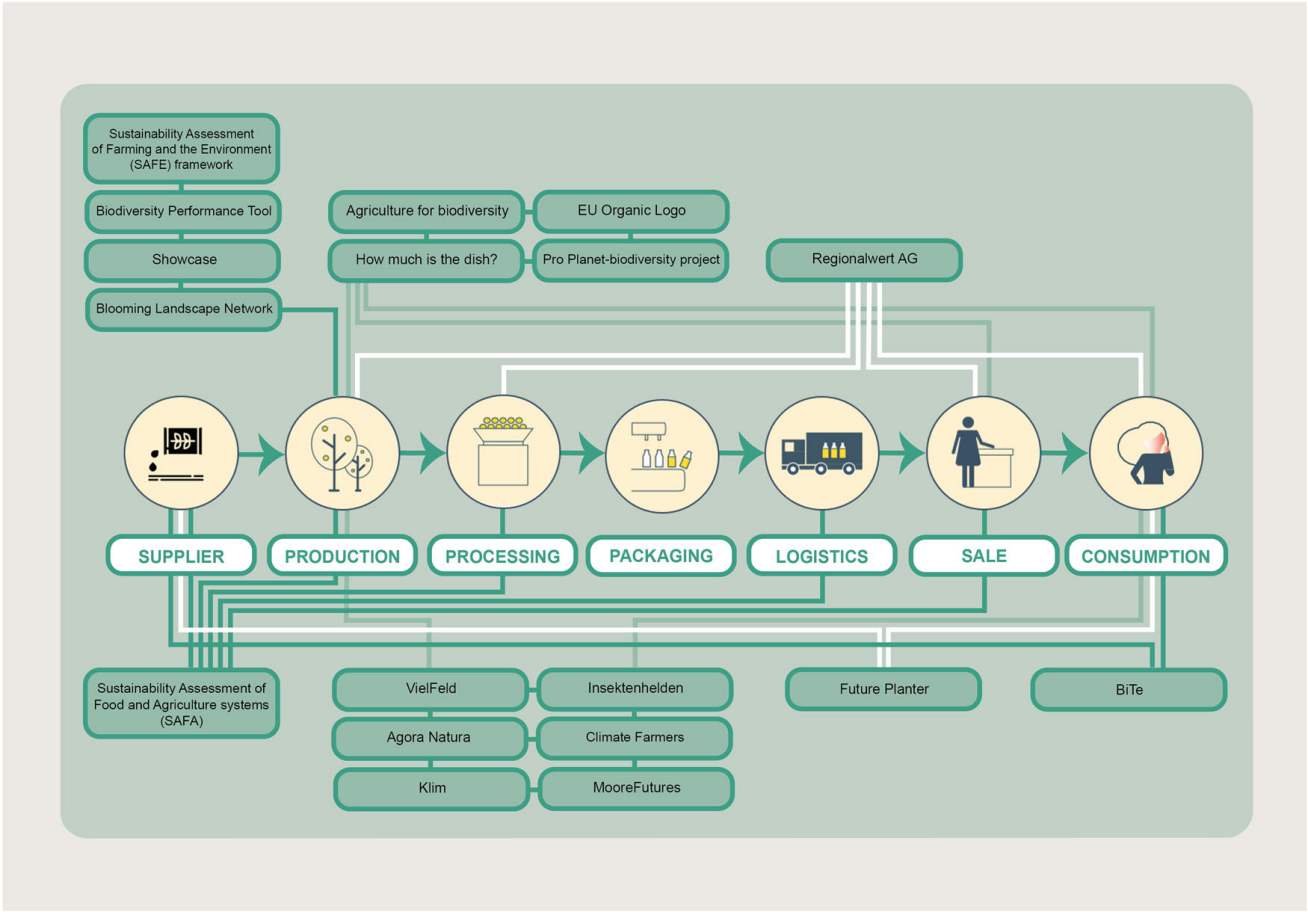
Overview of the valorization options and their connections to various stages of the agri-food value chain

Figure 5 shows valorization options can be linked to different stages of the agri-food value chain. Some options only address one or two stages, e.g., “Vielfeld”, “Insektenhelden” and “Agora Natura” approach the production and consumption stage, whereas other options address several stages, such as “Regionalwert AG” which addresses production, processing, logistics and consumption.

### Viability of the Valorization Options in the Four Future Scenarios

In Table 5, the viability of the valorization options in the different future scenarios is presented. The color scale indicates if an option is more suitable (green colors scheme) or less suitable (red color scheme) in a specific scenario. This gives an overview of how robust the options are when confronted with different framework conditions. The presented valorization options were selected and scored during the workshop by the participants.

**Table 5:** Viability of the valorization options in the four future scenarios

The assessment of the future viability of valorization options shows that some options are more suitable in different scenarios, while others would only be applicable in one specific future. For the first cluster, the example of “Agora Natura” was selected, which fits in all the four presented scenarios. Especially platforms offering the promotion of BES fit well together with local framework structures as described in scenario A and B. Through the regional connection to these platforms, spatial and emotional connection is created. In scenarios describing a more globalized world, as in scenario C and D, this valorization option would be applicable, as a platform with an innovative concept (scenario C) or as a kind or regulatory sandbox (scenario D).

The token^11^ for tree planting “Treecycle” was assessed to be less suitable as the implementation would be hindered in scenario B, due to a lack of digitalization and reluctance to use digital technologies in general. The same assessment was made for scenario C with the explanation that the economic pressure in this scenario would not be high enough, as land is not owned by private persons. However, the unregulated market system in scenario A would allow to act on a local basis, while at the same time considering global welfare. In general, tokens are a means to increase transparency and therefore generally usable for futures requiring highly transparent information.

Within the category certification and labeling the option “agriculture for biodiversity” was assessed to be supported by the framework of scenario A, because the retailer is in close contact with the local food chain actors. Scenario B would only slightly promote this option as there is a small market share of biodiversity friendly products. While in scenario C and D the implementation of this option is hindered.

The valorization option “lark bread” would be promoted in scenarios A, B and C but hindered in scenario D, as there is a lot of economic stress on consumers and farmers not giving the freedom to act on biodiversity protection.

In the cluster Environmental management/CSR the “SAFE” framework was assessed as one valorization option. A basic requirement for this option is the availability of data, which can be met in all scenarios except scenario B. Especially within scenario C and B, the availability of centralized data collected by the state or dominant actors within the agri-food value chain support the application of the “SAFE” tool. The same logic could also be applied to other data-intensive valorization options.

Using the example of “eco-dots”, scenario A, with a strong influence of retailers on prices, quality and product lines, as well as production conditions, and scenario C, where agriculture belongs to the state and a common good economy is pursued, the framework of tradable permits and quotas is supported.

The implementation is hindered in scenario B, because the missing information flow is not ideal for the trade with permits. Meanwhile, in scenario D, the retailer could act as the certificate purchaser leading to slight support of this option.

## Discussion

Our assessment contributes to the identification of valorization options of BES along the agri-food value chain and the classification into four clusters using different mechanisms of valorization. Besides the legal assessment, the assessment using the DPSIR framework showed that valorization options analyzed in a systemic way can be better adapted or understood. In addition, the options for the valorization of BES have to be assessed according to their future viability in alternative futures.

Governments are becoming more aware of the significance of BES and the challenges it faces, but public options have shown to be ineffective in adequately tackling threats to BES. This is because governments are often limited by a lack of resources (e.g., funding) as well as administrative and political barriers. The focus of the article was on private valorization options, as they can be complementary to public options but are often overlooked in scientific literature. Public options, such as the agricultural greening measures included in the European CAP framework, are either voluntary or obligatory measures leading to different payments or incentives to farmers. As they are set by public authorities, these measures are large in scope. They can contribute toward valorizing BES, but because they must be generally applicable and focus primarily on the production stage, they are unable to exploit the individual characteristics of the many actors in the agri-food value chain. Private options, on the other hand, are flexible in their application and have the advantage of being more targeted as well as engaging more stakeholders. Further, by utilizing market-based incentives, private options do not face the same funding limitations as public options. However, as there is no overview or repository of private actions, it is difficult to get recognition for private valorization options as such and to get information about further options.

### Advantages of Combining Research on BES with Foresight Studies

Systemic thinking builds the interfaces from generic approaches toward concrete actions to preserve biodiversity, and closing the loop back from the individual perspectives towards the larger view on the entire food system (Levy et al. 2018). The transformation already taken place in different food systems shows that many different drivers can be influential (Moller et al. 2019; Reardon et al. 2019).

The question is if mechanisms can be found to ensure a sustainable food production that promotes BES without passing on the majority of physical and organizational burden to farmers? This concerns financial aspects as well as the trade-offs of such measures regarding the long-term decisions of agricultural businesses (e.g., the recovery of land used to plant hedges is not possible). The answer to the question if more biodiversity in agriculture with a higher degree of ecology is economically sustainable depends not only on the existence of higher compensation payments in the future, but also on the existence of private options to, for example, give consumers more incentive and influence for achieving a higher degree of BES. Current approaches of considering non-marketable goods such as BES in management decisions have to be expanded and supported by elements of valorizing these measures. However, the literature analysis revealed a lack of consideration of private valorization options within the scientific community. Overall, stronger market interventions are necessary and external effects have to be internalized to halt biodiversity loss. This could be operationalized analog to CO2 certificates, where a biodiversity price could be initiated. In this sense, public interventions will remain important and must be considered together with private approaches to achieve a significant improvements of biodiversity, as well as to bring biodiversity more into public discussion and enhance its visibility.

### Public Engagement and Economic Incentives via Markets for Voluntary Services

A general characteristic of the category Markets for voluntary services is that there is always an intermediary for the mediation between the involved parties in the implementation of an action. A significant advantage of options within this cluster is that larger sums of money are made readily available for direct planning and implementation. Nevertheless, it must be taken into account that these are voluntary payments for public goods and services rather than products for immediate use (Albert et al. 2017; Sattler and Matzdorf 2013). From practical experience, based on stakeholder feedback during the workshop, the disadvantage of voluntary markets is the non-continuous cash flows to finance measures with a long-term perspective. A possible solution would be to extend the options towards longer investment periods.

### Awareness Raising and Economic Incentives via Labeling and Certification

During the workshop, concerns were raised regarding labels, that most of the revenue generated by labels remain with the retailer, which points to doubts about “supply chain transparency” (Sodhi and Tang 2019). While labeling is associated with rules and costs for the producer, this has to be balanced with the achievable added value. The existing procedure is that labels prove a certain product characteristic and therefore the product is more expensive. However, biodiversity-friendly products could be cheaper than the conventional products, if true cost accounting was applied to conventional products (True Cost Initiative 2022). In order to provide a functioning market, the use of labels is limited by the principle of labeling integrity, which is the underlying concept of legal provisions referred to as food quality (Olbrisch 2022).

Among the general public, there is lack of knowledge regarding biodiversity and its meaning (Stampa and Zander 2022), or what external environmental costs arise due to certain production regimes (Michalke et al. 2022). Only 38% of Europeans are aware of what biodiversity is, which highlights the need for more awareness raising and education on BES (Renna 2015). Furthermore, labels should be transparent and trustworthy for consumers, however, if there is too much information on a product, as a result of cognitive limitations, consumers “use heuristics and rely on aggregated levels of information such as price or brand names as summary [...] constructs instead of becoming lost in the details” (Dörnyei and Gyulavári 2016).

### More Interface between Science and Business Sustainability Reporting

CSR has been criticized for its lack of success till now due to missing motivation for companies to adopt such approaches (Krause et al. 2021). Of companies that have incorporated biodiversity in their CSR practices, very few make tangible commitments that are ‘specific, time-bound and measurable’ (Addison et al. 2019). Without making measurable commitments, CSR reporting runs the risk of becoming an instrument for ‘green washing’, indicating its limit as a viable BES valorization option and a true driver of change (Gatti et al. 2019). As pointed out in the legal assessment, the non-legally binding nature of CSR approaches means there are no direct penalties associated with voluntary commitments to BES measures that are not fulfilled. However, as public awareness for BES increases, companies may be more inclined to fulfill their commitments to avoid public scrutiny.

As noted by the workshop participants, sustainability assessment tools, such as SAFA, SAFE and the biodiversity performance tool, face drawbacks in terms of the high data requirements for conducting such assessments. This particularly applies to small and medium sized companies, where obtaining the necessary data for conducting sustainability assessment would be costly in terms of time and money. However, implementing these tools may become easier through digitalization e.g., through sensor networks, UAVs, blockchain, which will allow for transparent data acquisition and collection on a continuous basis (Weersink et al. 2018). This was a view also expressed by participants in the workshop who saw greater potential for such options in future scenarios that exhibited higher degree of digitalization. In this sense, digitalization could be an important leveraging tool for valorizing BES.

It has been shown that biodiversity-oriented CSR may benefit from taking a value chain approach (Wolff et al. 2018b). This is especially true for companies that do not have a direct influence on biodiversity through their operations, but may have up- and down-stream influence in the supply chain. For instance, companies’ could employ Life Cycle Assessments (LCA) of its products as part of its CSR activities to identify where biodiversity is being impacted along the value chain, although there are currently few LCA approaches that integrate BES.

### On the Interface between Policy Instruments and Private Options

Tradable permits and quotas are relevant instruments for efficiently managing environmental problems. Due to their prevailing private contractual nature, private companies are important intermediaries to facilitate the actual trading activities. Within the agri-food system, private intermediaries are crucial players to facilitate BES valorization in the agri-food value chain (Voglhuber-Slavinsky et al. 2021). Such coordination is required in the valuation procedure (see Fig. 1), where the intermediary evaluates land quality with regards to its biodiversity potential, followed by a quantification procedure. As a subsequent step, the actual valorization is carried out in terms of an investment or payment of a certain land development project.

The more private companies as intermediaries emerge to coordinate these efforts, the more diverse compensation opportunities might develop in the future, not only for biodiversity, but also to broaden the spectrum of other ES that urgently need to be considered. Trading platforms in this context could help to reduce transaction costs.

### Legal Assessment

We found several public law requirements for the use of private options. For the first time the Taxonomy Regulation defines when economic activities are considered “ecologically sustainable” (Lamy and Bach 2020). This triggers the company’s obligation to inform investors on the companies contribution of their economic activities to environmentally sustainable operations (Dietz 2022). In regards to tokens or other blockchain applications for proof or certification purposes, it must be considered that these applications might infringe the right to be forgotten from Article 17 of the GDPR (Schöbel 2021). Particularly in the case of smaller agricultural businesses, the GDPR is applicable (Kipker and Bruns 2020), and therefore, the use of these technologies may exclude these companies from participating in the respective markets. Concerning labels, the use of them is limited by the principle of labeling integrity in order to prevent failure of market (Olbrisch 2022). CSR Measures can be regarded as a set of self-binding rules and cannot directly be enforced by legal means, but if regarded as advertising communication they can cause legal consequences in case of non-compliance and potentially interference with the market (Alber 2021).

Some of the valorization options can be supported by the legal means that are already available under the current circumstances. Other options might necessitate completely new legal instruments as a reaction to disruptive innovations in the rising era of digital agribusiness (e.g., blockchain-based token models).

With regard to the scenarios, a mix of options constructed by hard law and further “non-legally constructed” options might be realistic and appropriate. It would probably be the most efficient way of “BES – governance”. Taking into account this coexistence might have a significant potential for creative solutions of valorization concepts in the future.

### DPSIR Assessment

Our analysis showed that clusters of valorization options address specific elements in the DPSIR framework. For example, options within the CSR as well as the Labeling and certification cluster have strong connection to *Drivers*, especially in terms of awareness raising and shaping societal attitudes toward BES. To these ends, increasing consumer awareness and knowledge of BES through eco-labels should be viewed as important long-term strategy for valorizing BES (Teufel et al. 2021).

Options within the Tradeable permits and quotas cluster and Markets for voluntary services cluster most directly address *States* in the DPSIR framework. In both cluster, options are heavily reliant on quantification and monitoring, as measures that maintain ecosystem states are typically results-based. The feasibility of such valorization options presupposes proper monitoring mechanisms, a certain level of awareness for a particular BES as well as willingness to pay for its protection.

Using the DPSIR approach further demonstrate how the impact of BES feeds back into the valorization process by modifying the value of BES over time. For instance, it can be reasonably assumed via our analysis that that cumulative impacts of valorization option at different points in the DPSIR framework can effectively increase the provision of BES. However, in certain contexts, other options may be more effective than others. This necessitates an adaptive management perspective (Holling and Walters 1978) when designing and implementing valorization options.

We find the DPSIR approach a useful analytical tool for accounting the diverse ways valorization options can provide viable responses and solutions for promoting BES. However, since our DPSIR assessment is a generalization of the system dynamics affecting valorization options and BES, further research is needed to go deeper into the individual causal relationships of valorization options and elements in the DPSIR framework. Additionally, developing suitable indicators to use within this framework would facilitate empirical studies to more accurately investigate the impact of valorization options, thereby shedding more light on their efficacy.

### Limitations of BES Valorization Options

It would be worthwhile to explore options, methods or frameworks for the promotion of BES from other fields outside the agri-food sector. The applicability of options going beyond what is currently used could be hindered by the complexity of the valuation of BES, as there is no consensus on how it should be measured, unlike other topics such as carbon offsetting, which have established standards. (Verra 2022). The recently developed Planet-Score^12^ for example, works with an extended life cycle assessment capturing as well impacts on biodiversity of the labeled food products. Other examples are the labels from IP Suisse (IP-SUISSE 2022) and Bioland (Bioland e.V. 2022) who set minimum standards for biodiversity to enable the use of the labels. The disadvantage of introducing many different approaches is that it may contribute to consumer non-transparency and confusion as well as lead to non-standardized scientific quantification procedures, which compromises a joint approach.

Many of the valorization options summarized in this article are based on one-time payments, while the implemented measures are either only possible to implement over a longer period of time or have an increasing marginal utility over time. Therefore, regular payment flows would be more desirable. For example, investors to the Regionalwert AG (Regionalwert AG 2022) pay once for a share. This is more applicable to finance regional infrastructure than regular measures to protect biodiversity.

### Limitations of the Study and Reflections on the Method

It is important to acknowledge some limitations of our study. Firstly, it should be noted that our research does not aim to provide a comprehensive analysis of all existing private BES valorization options. To achieve that, a systematic review of both scientific and public sources would need to be conducted. Instead, our study offers a preliminary exploration of the topic by highlighting a variety of examples of private BES valorization options from Europe and Germany. Additionally, the proposed future scenarios are just some thinkable developments for the future, other scenarios could as well be used to evaluate valorization options. For example, scenarios might be tailored to a specific region or might integrate specific key factors useful for the analysis. Despite this, we consider scenarios to be a valuable tool for encouraging reflection and broadening the decision-making capacity of stakeholders in the context of our study.

During the workshop, it became apparent that an extended understanding of the agri-food value chain can be an advantage. Using the concept of a value network instead of a value chain can be enriching for further research to map connections and identify gaps where further linkages can be identified. In addition, the understanding of the agricultural system today is to merely produce food, feed and fiber, neglecting the other services which are pictured in the ecosystem service concept. As a consequence, the understanding of the agri-food system could be complemented and broadened by including biodiversity and ecosystem service aspects.

## Conclusion

The current agri-food system focuses on the production of food, feed, fuel and fiber using BES as unrewarded input factors. Public policy instruments designed to foster the protection and integration in decision making of these resources failed to achieve a significant improvement and should therefore be amended by private valorization options. This can be achieved by increasingly holding to account other actors along the different stages of the agri-food value chain alongside production. This does not mean to choose between public (governmental) or private valorization options, but to use both approaches in interplay for effective BES governance (Muradian and Rival 2013; Primmer et al. 2015). Regulations can provide a baseline to environmental protection, while private options, with voluntary and direct payments for BES facilitate achieving an actual investment goal (Sterner and Coria 2013; van Hecken and Bastiaensen 2010; Vatn 2010). The insights of this study show there is the need to account for different private valorization options to make them accessible to a broader audience. At the same time, they have to be contextualized concerning their legal requirements, their role in the wider system and their future viability. For example, private options can’t exist and operate in a detached autonomous legal sphere, as they not only need to comply with public law, but tend to interact with it.

Contextualizing valorization options within a wider socio-ecological system is necessary for understanding the impacts of such options and, also underlines the need for an adaptive governance approach. Depending on current and future system contexts, certain options may be more effective than others at promoting a particular type of BES, which means valorization options need to be reevaluated, reapplied, and perhaps even reinvented moving forward. As all valorization options need time to show impact, we suggest to account for changes in future framework conditions using scenarios.

## Appendix A: Description of the Scenarios “Agribusiness in 2035—Farmers of the Future”

The scenarios were developed in the project DAKIS^13^ (Digital Agricultural Knowledge and Information System) and explain the future environmental framework for the decision support system designed in the project. Detailed information on the scenario process as well as comprehensive descriptions can be found in (Dönitz et al. 2020). In the following a concise overview of the four different scenarios is given.

Scenario A Environmental Protection by Local Food Circles and Qualitative Growth describes a world with a high diversity of land owners as there are multiple possibilities for investment in agriculture. Within society a change in thinking has taken place and a qualitative growth paradigm is followed. Farmers are well perceived for their contribution to society. Retailers influence prices, quality, product lines and production conditions, they act as the information hub within the agri-food value chain. On the farm there is a mixture of large, manually operated machines and small autonomous robots.

Scenario B Event Consumption by Face-to-Face Interaction in Local Food Circles is characterized as well by a high diversity of land owners a qualitative growth paradigm within society. In addition, the farmer is appreciated for taking care of the natural landscapes. Different than in scenario A is however that there is resistance to digitalization. Consumers buy local or regional food and exchange information at the point of sale. Hence analog information prevails and this makes the exchange of information along the value chain more complex. Agricultural production is dominated by manually driven large machines and unrelated assistant systems. Therefore, the individual production steps are intelligent, but not connected to each other.

Scenario C Environmental Protection by Global High Tech and Regulation shows a differentiated picture then the first two scenarios. Agricultural belongs to the state and of land is conducted according to economy for the common good. Within society a strictly sustainable economic model prevails, products are used over a longer period of time and plant-based nutrition is preferred. Farmers are not visible within society and their contribution to landscape maintenance is not acknowledged. Their work is not well understood and at the same time there is low transparency of agricultural production. New technologies and the expansion of network coverage allow a seamless information flow along the value chain. Therefore artificial intelligence is as well integrated in everyday business of the farmers.

In scenario D Reduced Consumption and De-growth by Necessity farmers pay a high price for renting agricultural land, as there are only a few owners of land. Land grabbing a common practice in this future. As the title of the scenario explains society follows the paradigm of reduced consumption, but not out of conviction but by the necessity to do so. Farmers are perceived within society as businesses selling food, feed and fiber. Retailers have a very strong position within the value chain. They influence prices, quality, product lines and production conditions. Artificial intelligence, for example is used by them for intelligent pricing, as well as they apply it to collect customer profile data to maximize profits. Sensors are integrated in primary production and in every part of the value chain connecting all the stages from input supply to consumption and waste treatment.

## Appendix B: Detailed List of Valorization Options of BES

Tables 6–9

**Table 6:** Markets for voluntary services

Title	Short description	Primarily involved stages of the value chain	Financing	Mechanism (valuation and valorization)
CONTRACTS 2.0 Innovative contracts for farmers and nature https://www.project-contracts20.eu/	Contract 2.0 develops new contractual approaches to incentivize farmers to provide more environmental public goods in addition to private goods.	Production, consumption *Entire VC* Contracts 2.0 will investigate value chain approaches which are collaborative models to valorize environmental public goods within value chains and integrate the cost for environmental services in the product price.	Public and private	valuation: Result-based payments on the basis of indicators and quantification methods valorization: integration of the cost for environmental services into the product price
AGORA NATURA Nature conservation together and concretely https://agora-natura.de/	Online marketplace for certified nature conservation projects. Private investors and companies can specifically promote biodiversity and nature services through the purchase of nature conservation certificates.	Production, consumption (private individuals, companies) Farmers or landowners as recipients of monetary benefits, which are financed by consumers or businesses. Financing of biodiversity measures takes place through the purchase of certificates.	Private financing from individuals or companies	valuation: quantification of biodiversity and ES valorization: contract between private companies/persons and farmers with certification
REGIONALWERT AG We fight for farmers, bees and the soil https://www.regionalwert-leistungen.de/	It is an offer for citizens to actively take responsibility for their region and the local food supply in the form of a share purchase from their regional representation of the Regionalwert AG.	Production, processing, transport, consumption A more regional and intermediate value chain will be created by this initiative.	Private financing from individuals	valuation: value performance calculator (Leistungsrechner) valorization: share purchase for citizens (Bürgeraktie)
KLIM. Your companion for regenerative agriculture. The free platform for soil health & climate protection https://farms.klim.eco/	The app enables farmers to implement management measures to improve carbon sequestration. The farmers can obtain a certification and the products will be labeled as carbon farmed.	Production, consumption Payments come from the consumers for the products, from investors and in case of a public CO_2_ pricing scheme also from regulation.	Private financing from individuals, financing authorities, public entities	valuation: method-based approach, where implemented measures are paid for based on their potential benefit calculated applying scientific data valorization: payments from investors, consumers or public authorities
ECOCROWD. Crowdfunding for sustainable projects https://www.ecocrowd.de/en/	A platform, launched by the German Environmental Aid (Deutsche Umwelthilfe), where sustainable projects and start-ups are presented which need start-up assistance. Citizens can make a contribution to financing and will receive a reward.	Entire value chain Projects can be related to every step in the VC.	Private financing from individuals	valuation: dependent on the project valorization: payment for a project connected to various rewards
FUTURE PLANTER Improving our biodiversity with concrete measures https://futureplanter.ch/	The non-profit foundation wants to promote biodiversity and personal responsibility in Switzerland. The platform offers the possibility to find out which endangered wild bees and butterflies occur in one’s own environment and suitable native wild shrubs can be ordered.	Suppliers, consumption	Private financing from individuals, companies and communities	valuation: the implementation of the measure is done by individuals and will not be assessed valorization: payment by consumers
VIELFELD Let the fields bloom! https://viel-feld.de/	People can become a supporter of one of the blooming fields with a sponsorship. Customers can select a farm and a size for the flowering area, which they want to support.	Production, consumption	Private financing from individuals	valuation: payments for farmers to adopt measures valorization: payments from consumers
INSEKTENHELDEN https://insektenhelden.de/	The vision of the start-up is the first online platform for joint insect protection. The farmers offer an area to consumers, who can choose from various support options.	Production, consumption	Private financing from individuals, businesses, foundations	valuation: monitoring of measures and subsequent payments for farmers valorization: payments from consumers
BITE Biodiversity beyond the plate: projects to increase biodiversity in supply and demand in the out-of-home catering sector https://wupperinst.org/p/wi/p/s/pd/923	Development of a biodiversity index for lunchtime meals of the out-of-home catering industry. (Agro)biodiversity will be measured in order to make the influence of a lunch on (agro)biodiversity visible.	Gastronomy/caterers, consumption	Public funding by BMBF	valuation: measurement of biodiversity valorization: information on the impact on biodiversity of the different meals
MOORFUTURES, Paludiculture Agriculture and forestry on rewetted peatlands https://www.moorfutures.de/	Paludiculture promotes biodiversity and other ecosystem services of peatlands. It offers perspectives for agriculture and tourism in poorly developed regions.	Production, consumption	Private financing from individuals and companies	valuation: scientific monitoring of the climate impact as part of the project planning and every 5 years after the implementation of the rewetting of the peatland valorization: purchase of the MoorFutures carbon certificates
CLIMATE FARMERS Building the infrastructure to scale regenerative agriculture & reverse climate change. https://www.climatefarmers.org/	The potential of regenerative agriculture to store carbon in the ground and restore ecosystems, all while improving the livelihood of farmers is in the focus of the initiative. To enable more farmers to take the step into regenerative agriculture, financial instruments for the transition period are developed.	Production, consumption	Financing from companies	valuation: monitoring valorization: voluntary carbon market for companies
TREECYCLE Investments into eucalyptus trees by blockhain technology https://treecycle.ch/	Investors directly invest in eucalyptus trees by blockhain-driven security tokens (eTREEs).	Entire VC (all parties involved benefit via blockchain technology), all VC members can be investors	Private/monetary	valuation: each eTREE is legally linked to a real eucalyptus tree using a blockchain-driven security token valorization: voluntary investment in tree planting via tokens
BLOOMING LANDSCAPE NETWORK NETZWERK BLÜHENDE LANDSCHAFT – NBL https://bluehende-landschaft.de/bluehflaechen/landwirtschaft/	The Blooming Landscape Network focuses on the positive image of a blooming landscape for all pollinating insects.	Production	Private financing from individuals and companies	valuation: no valuation of BES valorization: direct payment for a measure (measure oriented), signs and info boards

The term consumption, not just refers to the consumption of food, but as well of BES

**Table 7:** Labeling and certification

Title	Short description	Primarily involved stages of the value chain	Financing	Mechanism
ECO-SCORE Sustainability labeling for food. https://docs.score-environnemental.com/	The Eco-Score evaluates the environmental characteristics of a food product within five levels. There are 16 categories determining the environmental footprint of a product and play a role in the product’s rating. The rating scale contains five levels from A to E. A food with an A-rating has a low impact on the environment and a product with an E-rating has a high impact on the environment.	Entire value chain	Private	valuation: Life Cycle Assessment valorization: labeling
PRO PLANET-BIODIVERSITY PROJECT Partnership at eye level. https://www.rewe-group.com/de/presse-und-medien/newsroom/stories/pro-planet-apfelprojekt-partnerschaft-auf-augenhoehe/ https://www.nabu.de/spenden-und-mitmachen/fuer-unternehmen/kooperationspartner/12437.html	Within the project more food and nesting opportunities are created for flower-visiting insects on the conventional acreage of apple growers. The products are labeled with the Pro Planet seal “For more biodiversity”.	Production, retail, consumption	Private	valuation: measure-oriented approach (implementation of biodiversity promoting measures): evaluation of site-suitability for measures, measure implementation and evaluation-based on scoring system, producer certificate in compliance with scoring system (regional level) valorization: certification and Pro Planet-Label „For more biodiversity“ on REWE Group products
AGRICULTURE FOR BIODIVERSITY https://www.landwirtschaft-artenvielfalt.de/	With the project a retailer, WWF and organic farming associations are working together for biodiversity. The retail group guarantees the participating farmers the purchase of their products and rewards the additional effort that results from the project measures.	Production, retail, consumption	Private	valuation: measure-oriented approach (implementation of biodiversity promoting measures): evaluation of site-suitability for measures, measure implementation and evaluation based on scoring system, producer certificate in compliance with scoring system (farm level) valorization: “Agriculture for biodiversity” Label on Edeka products
EU ORGANIC LOGO https://ec.europa.eu/info/food-farming-fisheries/farming/organic-farming/organic-logo_en	Only products for which an approved inspection body has certified that they have been produced organically may bear the organic logo. This means that they must meet strict conditions for production, transport and storage.	Production, retail, consumption	Private	valuation: compliance with cultivation and husbandry standards (control mechanisms included) valorization: labeling and certification
NATURLAND https://www.naturland.de/de/naturland/wofuer-wir-stehen/qualitaet/qs-richtlinien.html	Naturland standards follow a holistic approach: sustainable management, practiced nature and climate protection, safeguarding and preserving biodiversity, soil, air and water, and consumer protection are considered. In addition, the three pillars of sustainability support Naturland’s holistic concept.		Private	valuation: sustainability assessment valorization: labeling and certification
LARK BREAD https://www.lerchenbrot.de/	To reward the creation of lark windows in wheat fields, the regional production and marketing chain guarantees that the grain harvested in the lark fields reaches the store counter directly via the miller and baker - distinguished as “lark bread”.	Production, consumption Regional value chain	Private	valuation: measure-oriented approch (implementation of biodiversity promoting measures): evaluation of site-suitability for measures valorization: label “larch bread” indicating nature conservation and regionality; higher prices for the wheat for farmers
TEST MARK OF THE BIOSPHERE RESERVE SCHORFHEIDE-CHORIN https://www.schorfheide-chorin-biosphaerenreservat.de/hier-leben/pruefzeichen-des-biosphaerenreservates/	The biosphere reserve’s test mark is a cross-sector certificate that awards regional companies and products. The initiative gives entrepreneurs a platform to market their offers. The prerequisite for using the test mark is the adherence to the principles of the UNESCO biosphere reserve.	Entire value chain Nature conservation, local businesses (as well apart from the agri-food sector), consumption Farmers in the biosphere reserve do not use genetic engineering, test mark holders focus on preserving old varieties and thus a broad genetic base. The certification mark is a distinguishing feature for consumers when purchasing food products.	Private	valuation: sustainability assessment valorization: certification and label ling of products
HOW MUCH IS THE DISH? True Cost Accounting of environmental impact costs and “true price tags” in Germany www.homabile.de	In a campaign, a German supermarket implements two price tags for certain products. The conventional one shows the current market price and the other one displays the price including social and environmental cost.	Production, retail, consumption	currently no executed payments	valuation: quantification and true cost accounting valorization: awareness raising (price tag, not executed)

**Table 8:** Environmental management/CSR

Title	Short description	Primarily involved stages of the value chain	Financing	Mechanism
SHOWCASE SHOWCASing synergies between agriculture, biodiversity and ecosystem services to help farmers capitalizing on native biodiversity https://showcase-project.eu/	The research project is testing incentive models to encourage better management of biodiversity on farms.	Production	no direct financing/ no monetary valorization	valuation: quantification of biodiversity and ES valorization: EU GAP 2nd pillar funding, considering labeling in the future
BIODIVERSITY IN STANDARDS AND LABELS FOR THE FOOD SECTOR https://www.business-biodiversity.eu/en/welcome	The European Business and Biodiversity Campaign (EBBC) is a partner consortium which supports companies from all industries in integrating biodiversity into their corporate management. The key project LIFE Food & Biodiversity, funded by the EU LIFE program, aims to improve the biodiversity performance of standards and labels within the food industry.	Standard-setting organizations, retail and processing are the main focus	no direct financing/ no monetary valorization	valuation: defining relevant criteria JOE valorization: improving the performance of standards and labels
ENTERPRISE BIODIVERSITY 2020 (UNTERNEHMEN BIOLOGISCHE VIELFALT 2020) http://www.biologischevielfalt.de/fileadmin/NBS/documents/UBI/UBi-Basispapier_NF5_barrierefrei.pdf	Provides a forum for environmental ministries, NGOs, and companies to network and cooperate with the goal to give companies advice on how to reduce their impacts on biodiversity.	Entire value chain (minus consumers, plus standard-setting organizations and finance)	no direct financing/ no monetary valorization	valuation: specific to each company valorization: awareness raising and networking between enterprises
BIODIVERSITY PERFORMANCE TOOL https://bpt.biodiversity-performance.eu/	The objective is to identify and assess the state of biodiversity potential on a farm in order to propose an action plan to reduce the impact on the production system, conserve and enhance biodiversity by proposing sustainable measures.	Production	no direct financing/ no monetary valorization	valuation: sustainability assessment valorization: awareness raising for the farmers and promotion of sustainable measures on farm
SUSTAINABILITY ASSESSMENT OF FARMING AND THE ENVIRONMENT (SAFE) FRAMEWORK http://www.trifolium.org/beratung/beratung-von-unternehmen-organisationen/selbstbewertung-nachhaltigkeitschecks/safe-sustainability-assessment-for-enterprises.html	This tool proposes a consistent and comprehensive framework of principles, criteria and indicators for sustainability assessment of agricultural systems. Three spatial levels (parcel level, farm level and a higher spatial level like the landscape, the region or the state) can be assessed.	Production	no direct financing/ no monetary valorization	valuation: sustainability assessment valorization: awareness raising and promotion of sustainable measures on farm
SUSTAINABILITY ASSESSMENT OF FOOD AND AGRICULTURE SYSTEMS (SAFA) https://www.fao.org/nr/sustainability/sustainability-assessments-safa/en/	This tool is an universal framework for Sustainability Assessment of Food and Agriculture systems developed by the FAO. The SAFA guidelines, indicators and tool and the app are free to use.	supplier, production, processing, distribution, retail	no direct financing/ no monetary valorization	valuation: sustainability assessment valorization: awareness raising and promotion of sustainable measures on farm/enterprise
BIODIVERSITY IN GOOD COMPANY BUSINESS AND BIODIVERSITY INITIATIVE https://www.business-and-biodiversity.de/	In the initiative, companies from numerous industries have joined forces to work together to protect and sustainably use global biodiversity. In doing so, they contribute to the Convention on Biological Diversity (CBD). The aim is to halt the dramatic loss of ecosystems, species and genetic diversity.	Integration of biodiversity and ecosystem services into the sustainability management of Companies	no direct financing/ no monetary valorization	valuation: actions and aims are set individually by the companies, assessment depending on the options valorization: awareness raising

**Table 9:** Tradable permits and quotas

Title	Short description	Primarily involved stages of the value chain	Financing	Mechanism
ECODOTS We develop ecopoints in Schleswig-Holstein for private developers, communities and municipalities. https://www.ecodots.de/	If an enterprise receives the requirement to create area compensation due to construction measures, the initiative can support with the developed ecopoints. Property owners, in turn, can earn money by making their property area available to us for ecological upgrading.	Production If a compensation takes place, it will be implemented e.g., on a field and together with farmers Entire value chain Project developers/financiers along the value chain	Private/monetary	valuation: evaluation of land quality with regards to its biodiversity potential; quantification into ecodots valorization: area compensation is payed by project developers, “ecodots” is coordinating the compensation procedure
BRANDENBURG AREA AGENCY (Flächenagentur Brandenburg) https://www.flaechenagentur.de/	Flächenagentur Brandenburg GmbH is a nature conservation service provider and partner for investors, authorities, land users and nature conservation. They implement effective and long-term nature conservation projects throughout Brandenburg - land pools. With these, a diverse range of compensation measures is available in all natural areas of Brandenburg.	Production If a compensation takes place, it will be implemented e.g., on a field and together with farmers Entire value chain Project developers/financiers along the value chain	Private/monetary	valuation: evaluation of the land suitability for compensation measures for land purchase; full cost calculation of measure implementation valorization: “Investors legally obliged to compensate for impacting on nature and landscapes pay the Flächenagentur for areas held in reserve and any compensation measures implemented” (Matzdorf et al. 2014)

## Footnotes

1 Please consult section ‘Theoretical background’ Background for definition on the terms valuation and valorization.

2 Digital Agricultural Knowledge and Information System (DAKIS) https://adz-dakis.com/en/

3 The strategy describes various actions to be taken to promote biodiversity by for example extending protected areas and reduce the use of pesticides. The degradation of ecosystems is addressed as well. Commitments are set in order to achieve the strategy’s aims.

4 Compare Federal Administrative Court, Judgment from April 27th 1984 - 1 C 10/84.

5 Regulation (EU) 2020/852 of the European Parliament and of the Council on the establishment of a framework to facilitate sustainable investment, and amending Regulation(EU) 2019/2088.

6 The differentiation between a label set by a private initiative and a label set by a public authority is based on who is the holder of the label and how the terms of use are statuated. e.g., in case that a public authority holds the concerning trademark and that the terms of use are statuated by law, it is justified to characterize such a label as ‘public’. In case that the corresponding trademark is held by a private initiative or company, the terms of use are usually arranged as license agreements or via trade mark statutes. Consequently these labels can be considered as ‘private labels’ (Olbrisch 2022).

7 Whereas individual trademarks are ordinated to an individual subject (i.e., for example, a farmer or a food company), collective trademarks are ordinated to a group of subjects (i.e., for example a group of food processing companies).

8 cf. Art. 5 I, II, 18 I, 44 I QualitätsVO, Art. 78 GMO-VO, Art. 1, 26 III, 36 LMIV, § 11 LFGB und § 8 II Nr. 4 MarkenG.

9 CSR goes beyond compliance with applicable law (Spindler 2019). A company can commit to compliance with its own standards but the law sets the minimum standards for this, because companies are not allowed to undercut the statutory minimum requirements through their own regulations (Walden 2020).

10 German Federal Nature Conservation Act (Bundesnaturschutzgesetz)

11 Tokens are an unembodied sequence of related characters. They stand for arbitrary information, for example a monetary value or the ownership of an object (Guggenberger 2021). The terms token and cryptocurrency overlap. Any form of cryptocurrency can also be called a token, but the term token is broader. Tokens can also represent a claim to certain goods, products, services or business shares, or they can represent securities (Kumpan 2020; Möllenkamp and Shmatenko 2019). The goal of token generation in this context relates in particular to harnessing transparency along value chains and allows a transparent tracking of measure.

12 The Planet Score goes further than the Nutri Score and the Eco score as it factors in pesticides, biodiversity and animal welfare. (see https://www.planet-score.org/)
